# Molecular cytogenetic characterisation of a novel *de novo* ring chromosome 6 involving a terminal 6p deletion and terminal 6q duplication in the different arms of the same chromosome

**DOI:** 10.1186/s13039-017-0311-y

**Published:** 2017-03-23

**Authors:** Nikolai Paul Pace, Frideriki Maggouta, Melissa Twigden, Isabella Borg

**Affiliations:** 1 Centre for Molecular Medicine and Biobanking, Faculty of Medicine and Surgery, University of Malta, Msida, Malta; 2 Wessex Regional Genetics Laboratory, Salisbury NHS Foundation Trust, Salisbury, UK; 3 Department of Pathology, Faculty of Medicine and Surgery, University of Malta, Msida, Malta; 4 Department of Pathology, Medical Genetics Unit, Mater Dei Hospital, Msida, Malta

**Keywords:** Ring chromosome 6, Molecular cytogenetics, Dysmorphology, Renovascular disease, FOXC1 gene

## Abstract

**Background:**

Ring chromosome 6 is a rare sporadic chromosomal abnormality, associated with extreme variability in clinical phenotypes. Most ring chromosomes are known to have deletions on one or both chromosomal arms. Here, we report an atypical and unique ring chromosome 6 involving both a distal deletion and a distal duplication on the different arms of the same chromosome.

**Case presentation:**

In a patient with intellectual disability, short stature, microcephaly, facial dysmorphology, congenital heart defects and renovascular disease, a ring chromosome 6 was characterised using array-CGH and dual-colour FISH. The *de*-*novo* ring chromosome 6 involved a 1.8 Mb terminal deletion in the distal short arm and a 2.5 Mb duplication in the distal long arm of the same chromosome 6. This results in monosomy for the region 6pter to 6p25.3 and trisomy for the region 6q27 to 6qter. Analysis of genes in these chromosomal regions suggests that haploinsufficiency for *FOXC1* and *GMDS* genes accounts for the cardiac and neurodevelopmental phenotypes in the proband. The ring chromosome 6 reported here is atypical as it involves a unique duplication of the distal long arm. Furthermore, the presence of renovascular disease is also a unique feature identified in this patient.

**Conclusion:**

To the best of our knowledge, a comparable ring chromosome 6 involving both a distal deletion and duplication on different arms has not been previously reported. The renovascular disease identified in this patient may be a direct consequence of the described chromosome rearrangement or a late clinical presentation in r(6) cases. This clinical finding may further support the implicated role of *FOXC1* gene in renal pathology.

## Background

Constitutional ring chromosomes are rare sporadic chromosomal abnormalities and can involve any of the 22 pairs of autosomes as well as the sex chromosomes. They arise from breaks in the two arms of a chromosome, followed by fusion of the two broken ends or of one broken end with the opposite telomere region [1]. Alternatively, two dysfunctional telomeres from the same chromosome fuse, resulting in the formation of a complete ring with no deletions. The clinical phenotype associated with ring chromosomes is highly variable, depending on the extent of the deletion and the chromosome involved.

Ring chromosome 6 is a rare sporadic chromosomal abnormality, first described by Moore et al in 1973 in a female infant who exhibited varying dysmorphic features [2]. The phenotypic features are very variable and range from an almost normal phenotype to severe malformations and intellectual disability. Peeden et al reviewed the variability of phenotypic features in 14 cases of ring chromosome 6 [3]. The most frequent clinical features include failure to thrive, congenital heart defects, intellectual disability, microcephaly and facial dysmorphology [4]. Also reported are various abnormalities in the ocular, auditory and central nervous systems.

We report a unique *de novo* ring chromosome 6 characterised by array-CGH and FISH involving a deletion in the distal short arm and a duplication in the distal long arm of the same chromosome.

## Case presentation

The proband was referred to the genetics clinic at the age of 49 years, during a hospital admission for a chest infection. He is the 4^th^ in a sibship of 5 offspring of non-consanguineous Caucasian parents. He was born by normal vaginal delivery following an uneventful term pregnancy. At birth he was found to have a small atrial septal defect. He was a slow feeder and early motor, cognitive and developmental milestones were delayed. At the age of 10 years he was diagnosed with bilateral hearing loss which worsened progressively. A CT scan of the brain performed at the age of 21 years, revealed the presence of a Dandy Walker variant, normal internal auditory *maeti* and a normal *sella turcica*. His medical history also includes chronic venous insufficiency and hypertension. The parents gave no relevant family history.

Physical examination at the genetics clinic revealed an adult male with moderate intellectual disability, short stature (height of 141 cms) and microcephaly (head circumference of 51.6 cms). Dysmorphic features included a right-sided hair whorl, a small nose, a high arched palate, a nuchal hump, mild scoliosis, brachydactyly and overlapping toes on the left foot. The skin was very dry and there was considerable venous insufficiency of the lower limbs.

An echocardiogram revealed borderline concentric left ventricular hypertrophy, mild right ventricular hypertrophy, a patent *foramen ovale* and a very small atrial septal defect, for which he subsequently underwent cardiac surgery. Blood investigations revealed renal pathology but renal imaging was unremarkable. Subsequently a renal biopsy revealed long-standing renovascular disease but no vasculitis.

### Methods

Array comparative genomic hybridisation (array-CGH) was performed at the Wessex Regional Genetics Laboratory using an Oxford Gene Technology (OGT, Oxford, UK) Cytosure^TM^ Constitutional v3 custom 8x60K oligo array, manufactured by Agilent Technologies [Agilent Technologies, Santa Clara, CA].

Dual-colour fluorescence in situ hybridisation (FISH) was performed with 2 bacterial artificial chromosomes (BACs) chosen from the Sanger 30K cloneset using the Ensembl genome browser (http://www.ensembl.org/Homo_sapiens/) together with the chromosome 6 specific centromere probe, D6Z1. The probes used were the 6p25.3 specific BAC, RP11-13J16 (GRCh37 bp 1296996 – 1357295) and the 6q27 specific BAC, RP11-417E7 (GRCh37 bp 169417007 – 16959015).

### Results

**Fig. 1:**
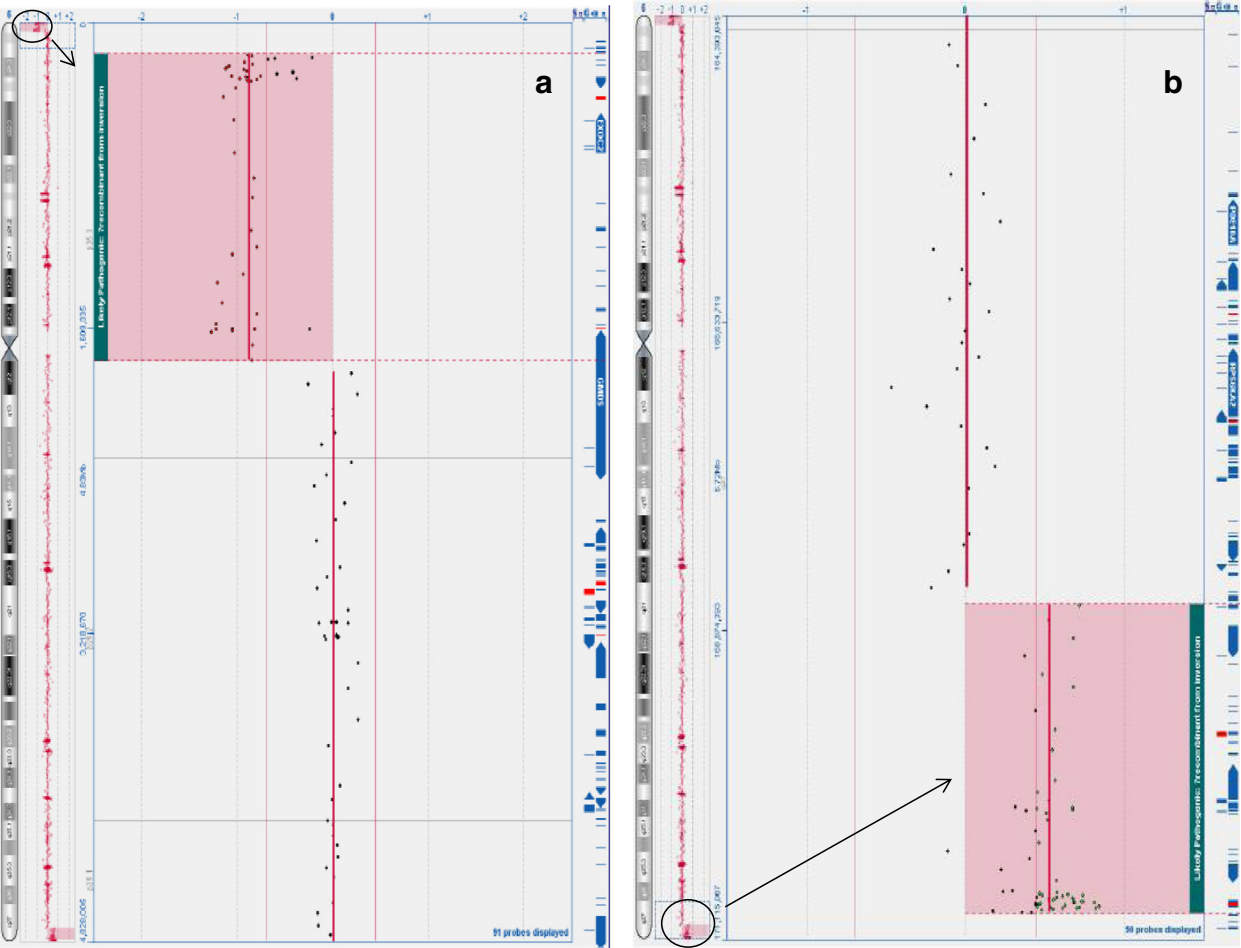
Array-CGH plots, analysed using Oxford Gene Technology (OGT) CytosureTM Constitutional v3 oligo array, showing. **a** the 1.8 Mb terminal 6p25.3 deletion with a log2 ratio of -1.0 at genomic coordinates 164360_1773695 (NCBI build 37, February 2009, hg19) and (**b**) the 2.5 Mb 6q27 duplication with a log2 ratio of +0.58 at genomic coordinates 168682830_170923504 (deleted/duplicated regions circled on chromosome 6 ideogram and magnified and highlighted in main image as indicated by an *arrow*)

Array CGH identified an approximately 1.8 Mb terminal deletion involving the distal short arm of a chromosome 6, with the breakpoint within a 68kb interval in 6p25.3 (arr(GRCh37) 6p25.3(164360_1773695)x1) (Fig. 1a). The array also detected an approximately 2.5 Mb duplication involving the distal long arm of a chromosome 6, with the breakpoint within a 125kb interval in 6q27 (arr(GRCh37) 6q27(168682830_170923504)x3) (Fig. 1b). The proband is, therefore, monosomic for the region 6pter to 6p25.3 and trisomic for the region 6q27 to 6qter.

To characterise the imbalances further and to determine whether they occurred in *cis* or *trans*, FISH studies were undertaken on peripheral blood metaphase chromosomes from the proband and both his parents using the 6p25.3 specific BAC RP11-13J16 and the 6q27 specific BAC RP11-417E7 as well as a 6 centromere probe (D6Z1).

**Fig. 2:**
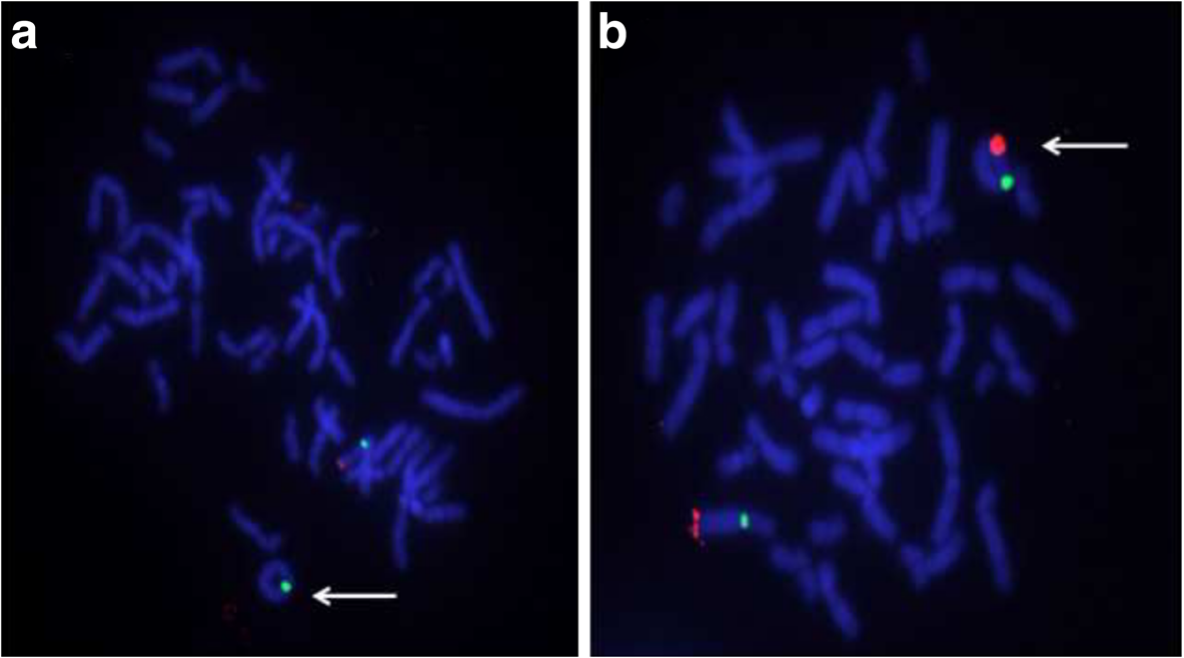
FISH with the chromosome 6 specific centromere probe D6Z1 (*green signal*), the 6p25.3 BAC RP11-13J16 (**a**; *red signal*), and the 6q27 BAC RP11-417E7 (**b**; *red signal*) showing a monocentric r(6) chromosome (*arrow*) with (**a**) 6p deletion and (**b**) 6q duplication

FISH confirmed both the 6p deletion and 6q duplication identified by array-CGH and showed that the imbalances are present in the form of a ring chromosome 6, r(6), which replaces a normal chromosome 6. The r(6) was shown to be monocentric, deleted for the short arm of 6p (6p25.3) and duplicated for the distal long arm of 6q (6q27) (Fig. 2). This r(6) represents an atypical and unique ring chromosome in that the duplicated material is located in a different arm of the chromosome to that of the deleted material. FISH studies on both parents showed that they had two normal chromosomes 6 and therefore the r(6) had arisen *de novo* in the proband.

## Discussion

Ring chromosome 6 is an exceptionally rare cytogenetic rearrangement that usually arises *de novo* and is associated with extreme inter-individual variability in clinical phenotypes. A number of reports have described the clinical features in r(6) patients. However, to our knowledge this is the first case of r(6) involving both a distal 6p deletion and a distal 6q duplication.

At least two case reports have described a r(6) involving a comparable deletion at 6p25.3 [4, 5]. Both patients had psychomotor delay, cerebral ventriculomegaly, a prominent forehead and malformed ears. Furthermore, Zhang et al report a deletion of identical size to the present case (1.78Mb at 6p25.3) and additional clinical features that include, microcephaly, hydrocephalus, epilepsy and hearing loss [5].

Submicroscopic deletions involving the 6p25 subtelomeric region is a distinct clinical syndrome. The clinical phenotypes described include developmental delay, intellectual disability, language impairment, hearing loss, and various ophthalmic, cardiac and craniofacial anomalies [6]. As the clinical phenotypes observed in ring chromosomes overlap with those of deletion of one or both ends of the respective chromosome syndromes, we explored karyotype-phenotype associations by reviewing literature that describes the function of genes located in the 6p25 and 6q27 regions.

A number of genes located in the 6p25-pter deletion interval have OMIM entries, namely *DUSP22*, *IRF4*, *EXOC2*, *HUS1B*, *FOXQ1*, *FOXF2*, *FOXCUT*, *FOXC1* and *GMDS*, as well as a number of uncharacterised genes. Of these, *IRF4* and *FOXC1* are human disease genes. Interferon regulatory factor 4 (*IRF4*) is a transcription factor essential for the development of T helper 2 (Th2), Th17 and Th9 cells, and the rs872071 variant in its 3-prime untranslated region is a susceptibility locus for chronic lymphocytic leukemia [7]. *IRF4* is also required for hair, skin and eye pigmentation [8].

The forkhead gene cluster at the 6p25.3-pter region includes *FOXQ1*, *FOXF2*, *FOXCUT* and *FOXC1* genes. These transcription factors are essential for development and morphogenesis. *FOXC1* and *FOXC2* genes are expressed during cardiac development, and mutations in these genes have been associated with a wide range of cardiac abnormalities [9]. Zhang et al suggest that the congenital cardiac anomalies observed in r(6) is due to haploinsufficiency of *FOXC1* [5].

Developmental delay and varying degrees of neurological defects are consistent features in 6p25 deletion syndromes. *FOXC1*, *FOXF2* and *GMDS* are involved in central nervous system development and function. Animal studies have shown that mice homozygous for a null allele of *Mf1* (the murine homolog of *FOXC1*) show congenital hydrocephalus and eye defects [10]. Haploinsufficiency for *FOXC1* has been associated with hydrocephalus in humans [6]. Abnormalities in the posterior cranial fossa, including Dandy-Walker malformation, mega-cisterna magna and cerebellar vermis hypoplasia have also been associated with homozygous *FOXC1* mutations [11]. Any deviation from normal *FOXC1* gene dosage results in central nervous system (CNS) vascular anomalies. Similarly, *FOXQ1* gene mediates neurite growth and neuronal differentiation [12]. *FOXF2* is expressed in murine pericytes, and *FOXF2* knockout mice develop intracranial hemorrhage, perivascular oedema and a leaky blood-brain barrier, further highlighting the role of these transcription factors in CNS development and function [13].

The *GMDS* gene encodes GDP-mannose 4,6-dehydratase, an enzyme that catalyzes the first step in protein fucosylation. This is an essential post translational modification in members of the Notch family of transmembrane receptors [12]. Notch signaling is essential for neuronal and glial cell differentiation, maturation, learning and memory. Song et al have described a zebrafish model that harbours a missense mutation in *GMDS* resulting in defective fucosylation of Notch receptors that leads to defects in neuronal development and synapse branching [13]. These studies strongly suggest that the varying degree of intellectual disability observed in r(6) cases is due to haploinsufficiency of the *FOXC1* and *GMDS* genes. Further evidence supporting the role of these two genes in neuronal function is provided by Hockner et al [14]. Here, the authors describe a case of *de*-*novo* r(6) in a 25-year-old female with short stature and only minor dysmorphic features, but with normal psychomotor development. Cytogenetic analysis had identified a 6p breakpoint telomeric to the *DUSP22* gene, with no disruption of either *FOXC1* or *GMDS* coding sequences at 6p. The short stature in this case report was attributed to mitotic instability of the ring chromosome.

The 6q duplication interval contains at least ten OMIM genes, of which four have OMIM Morbid entries (*SMOC2*, *THBS2*, *ERMARD* and *TBP*). Burnside et al report extensive CNS developmental abnormalities in a two-month old infant with a duplication of *THBS2*. This gene encodes thrombospondin 2, an astrocyte-secreted protein essential for synaptogenesis and neurite growth [15].

The r(6) outlined in this report is atypical in that no functional genetic material from the long arm has been lost, but material from the distal long arm (6q27) has been duplicated. Guilherme et al studied breakpoints and mechanisms of ring formation in fourteen cases of ring chromosomes, and reported two cases of ring chromosome 13 where terminal deletions of 13q were associated with duplications near the breakpoints on the same chromosome arm [1]. Similarly, Knijnenburg et al describe a case of ring chromosome 14 shown to have a terminal 14q32.33 deletion and an inverted duplication of 14q32.12 to 14q32.32 [16]. Comparably, Rossi et al investigated 33 probands with ring chromosomes using both array-CGH and FISH, and identified seven cases where duplications were also detected in ring chromosomes 13, 15, 18, 21 and 22 [17]. It is unusual for the duplicated material to be located on a different arm of the chromosome to that of the deletion. No similar findings involving ring chromosome 6 have been reported in the literature. However, additional variants and imbalances of ring chromosome 6 have been described. In a male infant with a number of dysmorphic features and a large patent ductus arteriosus, Lee et al report a mosaic karyotype with a dicentric ring chromosome (46, XY, r(6)(p25q27)/46, XY, dic r(6;6)(p25q27;p25q27) [18]. Birnbacher et al report a r(6) with an 6q26-qter deletion and no 6p subtelomeric deletion, and Nishigaki et al a similar r(6) with a 1.5Mb deletion at 6q27 [19, 20].

The clinical features described in this proband, in particular the cardiac and neurological anomalies are largely in common with other case reports of r(6). Haploinsufficiency for the forkhead gene cluster at 6p appears to be the major driver leading to development of these features. Of particular interest is the renovascular disease finding reported in this proband. While *FOXC1* disruption has been implicated in congenital anomalies of the kidney and urinary tract in both animal and human models, there is no reported link between this gene and the development of renovascular disease [21]. Renovascular disease was not described in any of the published r(6) patients. This may suggest that either this is a direct consequence of the unique r(6) presented here or that this is a late onset clinical feature. It may therefore be advisable to screen r(6) patients for renal pathology.

## Conclusion

This report provides a detailed characterisation of a novel r(6), involving a distal deletion at 6p25.3 and a distal duplication at 6q27. The learning disability, hearing loss, cardiac and CNS defects observed in the proband, can be attributed to hemizygous expression of *FOXC1*, *FOXF2* and *GMDS* genes on 6p25, and possibly also to the partial duplication of the distal 6q region. Screening of r(6) patients for renal pathology is advisable not only for the medical management of these individuals, but also to further explore the possible role of *FOXC1* gene in renovascular disease and secondary hypertension. Despite the genomic associations outlined in the literature, the correlation with phenotypes and clinical severity in r(6) cases remains highly variable and complex.

## Abbreviations

Array-CGH: Array comparative genomic hybridisation
BAC: Bacterial artificial chromosome
CNS: Central nervous system
CT scan: Computed tomography
FISH: Fluorescence in situ hybridisation
OMIM: Online Mendelian Inheritance in Man
R(6): Ring chromosome 6
